# Optimization of *Yarrowia lipolytica-*based consolidated biocatalyst through synthetic biology approach: transcription units and signal peptides shuffling

**DOI:** 10.1007/s00253-020-10644-6

**Published:** 2020-05-02

**Authors:** Ewelina Celińska, Monika Borkowska, Paulina Korpys-Woźniak, Monika Kubiak, Jean-Marc Nicaud, Piotr Kubiak, Maria Gorczyca, Wojciech Białas

**Affiliations:** 1 Department of Biotechnology and Food Microbiology, Poznan University of Life Sciences, ul. Wojska Polskiego 48, 60-627 Poznań, Poland; 2 INRA-AgroParisTech, UMR1319, Team BIMLip: Integrative Metabolism of Microbial Lipids, Micalis Institute, Domaine de Vilvert, 78352 Jouy-en-Josas, France

**Keywords:** Yeast strain design, Non-pretreated starch, Consolidated bioprocessing, Complex biopolymer

## Abstract

**Abstract:**

Nowadays considerable effort is being pursued towards development of consolidated microbial biocatalysts that will be able to utilize complex, non-pretreated substrates and produce valuable compounds. In such engineered microbes, synthesis of extracellular hydrolases may be fine-tuned by different approaches, like strength of promoter, type of secretory tag, and gene copy number. In this study, we investigated if organization of a multi-element expression cassette impacts the resultant *Yarrowia lipolytica* transformants’ phenotype, presuming that different variants of the cassette are composed of the same regulatory elements and encode the same mature proteins. To this end, *Y. lipolytica* cells were transformed with expression cassettes bearing a pair of genes encoding exactly the same mature amylases, but fused to four different signal peptides (SP), and located interchangeably in either first or second position of a synthetic DNA construction. The resultant strains were tested for growth on raw and pretreated complex substrates of different plant origin for comprehensive examination of the strains’ acquired characteristics. Optimized strain was tested in batch bioreactor cultivations for growth and lipids accumulation. Based on the conducted research, we concluded that the positional order of transcription units (TU) and the type of exploited SP affect final characteristics of the resultant consolidated biocatalyst strains, and thus could be considered as additional factors to be evaluated upon consolidated biocatalysts optimization.

**Key Points:**

*• Y. lipolytica growing on raw starch was constructed and tested on different substrates.*

*• Impact of expression cassette design and SP on biocatalysts’ phenotype was evidenced.*

*• Consolidated biocatalyst process for lipids production from starch was conducted.*

**Electronic supplementary material:**

The online version of this article (10.1007/s00253-020-10644-6) contains supplementary material, which is available to authorized users.

## Introduction

Efficient biorefining and bioprocessing rely on exploitation of renewable substrates, like residual biomass, which in its raw, non-pretreated form is mainly composed of complex biopolymers. Decomposition of complex substrates requires orchestrated action of several enzymatic activities. Depending on their overall amount, activity, and relative ratio, the process may progress with different efficiency. Nowadays, considerable effort is being pursued towards development of consolidated microbial biocatalysts that will be able to utilize complex, non-pretreated biopolymers and produce valuable compounds. While in some specific cases, native metabolism of a microbe can ascertain both growth on complex substrate and production of desired biomolecules; in many cases, one of the two obligatory qualities needs to be engineered. In such engineered microbes, specific synthesis of extracellular hydrolases decomposing complex substrates may be fine-tuned by different approaches, like manipulation with strength of regulatory elements governing transcription of the target genes, selection of optimal fusion with signal peptide (SP) that lead nascent polypeptides for secretion, and amplification of copy number of the recombinant genes or their localization in transcriptionally active locus in the genome. Nonetheless, it is known that the repertoire of approaches that enable adjusting metabolism of a microbe to become efficient consolidated biocatalyst does not end up on those classic strategies.

*Yarrowia lipolytica* is a non-conventional yeast species, which has long been applied as research and industrial workhorse in numerous processes, where its native or engineered metabolic qualities have been exploited (Nicaud 2012; Groenewald et al. 2014; Madzak 2018). Alongside the great interest that *Y. lipolytica* has received, a number of genetic engineering tools and protocols that are specific to this biological system have been developed (Madzak 2015; Larroude et al. 2018). Taking advantage of the progress within this field, it is now feasible to generate a number of variants of complex genetic constructions to carefully study and optimize their design, so that expression of target genes is adjusted to the requirements of a specific bioprocess. Due to unique native metabolic properties, like intrinsically high production of lipids (Tai and Stephanopoulos 2013), polyols (Rakicka-Pustułka et al. 2020), organic acids (Rywińska and Rymowicz 2010), and recently evidenced superior capacity for heterologous protein production over a typical workhorse in this regard—*Komagataella phaffi* (*P. pastoris*) (Theron et al. 2020), *Y. lipolytica* stains are frequently subjected to genetic modifications broadening its scope of utilized substrates, with the aim to improve economic feasibility of production processes (Ledesma-Amaro and Nicaud 2016). For example, *Y. lipolytica* strains have been endowed with artificial ability to grow on sucrose (Lazar et al. 2011), inulin (Rakicka et al. 2019), galactose (Lazar et al. 2015), starch (Ledesma-Amaro et al. 2015), or even cellulose (Guo et al. 2017a; Guo et al. 2017b). Engineering of renewable substrates utilization usually required introduction of several heterologous genes to *Y. lipolytica* cells, to either efficiently decompose biopolymer or provide a link between the new substrate and native metabolism of the host cell.

Upon introduction of several heterologous transcription units (TUs; a gene of interest flanked with regulatory elements) to a host cell, one can either use a set of separate DNA constructions bearing different genes of interest and selection markers (or rescueable marker) or use a multi-gene expression cassettes. The first strategy was indeed frequently followed upon metabolic engineering of *Y. lipolytica* for different purposes (Beopoulos et al. 2009; Dulermo and Nicaud 2011; Blazeck et al. 2014; Lazar et al. 2014; Lazar et al. 2015; Mirończuk et al. 2016), including previous report on construction of amylolytic strain (Ledesma-Amaro et al. 2015). Still, it is thought that multiple transformations, as required for the former strategy, may negatively impact the overall fitness of the transformed cells, which at some level may impose significant limitations to a given bioprocess. The latter strategy, relying on construction of a multi-gene expression cassette, also bears its limitations. It has been for example evidenced that individual gene expression becomes weaker in tandem genetic constructs composed of two heterologous genes (Wong et al. 2017). Still, with the progress in rapid cloning strategies, this approach gains importance. It has been pursued in *Y. lipolytica* for modulated synthesis of α-ketoglutaric acid (Holz et al. 2011; Otto et al. 2012), aroma compounds (Celińska et al. 2015b; Celińska et al. 2019) and lipids (Tai and Stephanopoulos 2013), glycerol utilization (Celińska and Grajek 2013) or heterologous synthesis of carotenoids and astaxanthin (Ye et al. 2012; Matthäus et al. 2014; Gao et al. 2014; Kildegaard et al. 2017), violacein (Wong et al. 2017; Wong et al. 2019), ω-3 eicosapentaenoic acid (Xue et al. 2013), flavonoids (Lv et al. 2019), and alpha-santalene (Jia et al. 2019). Organization of the multi-gene constructs with respect to the order and orientation of TUs is usually as follows: (1) dictated by availability of unique restriction sites, (2) random, or (3) organized according to their consecutive involvement in the native or synthetic pathways. However, growing evidence implies that genetic context and positional effects of a given TU may critically affect its expression (Bordes et al. 2007; Wong et al. 2017).

In the present study, we pursued optimization of *Y. lipolytica*-based consolidated biocatalyst. To this end, we used previously determined optimal fusions between signal peptides (SP) and polypeptides having amylolytic activities (Celińska et al. 2018), and organized them in tandem expression cassettes bearing two TUs. Further, we investigated if the order of TUs within the expression cassettes and type of SP in the pre-proteins’ impact expression of heterologous genes and the resultant strains’ characteristics. Amylolytic activities and starchy substrates were used as an easy to follow model, conferring strains with consolidated biocatalyst characteristics. Nevertheless, valorization of waste streams rich in starch that occur in massive amounts from confectionery manufacturing industries and bakeries, or equally as discarded, damaged, or out of date products that return on site, has been indicated as a so far ignored trend, with a huge potential for biotechnological valorization (Tsakona et al. 2014; Tsakona et al. 2019). Current waste treatments for these streams comprise animal feed, composting, or disposal in landfills, while could be used for “green” production of high-value added products through microbial transformations.

## Materials and methods

### Strains and routine culturing conditions

All bacterial and yeast strains used in this study are listed in (Electronic Supplementary Material, ESM Tab.S1. and Tab.S2). Cultivations required for molecular biology protocols followed standard protocols (Barth and Gaillardin 1996; Sambrook and Russell 2001). Briefly, *Escherichia coli* strains were cultured in LB medium (g/L 10, bacteriological peptone; 10, NaCl, 5; yeast extract; liquid or solidified with agar, 15) supplemented with appropriate antibiotic when necessary (ampicillin at 100 μg/L; kanamycin 40 μg/L), at 37 °C, 250 rpm in a rotary shaker incubator (Biosan, Riga, Latvia). *Y. lipolytica* strains were grown in YNB (g/L 5, ammonium sulfate; 1.7, YNB without AA; and ammonium sulfate; 20, glucose) or YPD (g/L 10, yeast extract; 20, bacteriological peptone; 20, glucose) media (liquid or solidified with agar, 15), at 30 °C, 250 rpm in rotary shaker incubator (Biosan, Riga, Latvia). Multiple strains were managed in microtiter plates (MTP); liquid cultures and sub-cultures were inoculated using stainless steel replicator (Sigma-Aldrich; Merck KGaA, Saint Louis, USA).

### Standard molecular biology protocols

Standard molecular biology protocols used in this study followed the methodologies described in Sambrook and Russell (2001). All oligonucleotides and longer synthetic DNA fragments used in this study are listed in Tab.S3. *E. coli* and *Y. lipolytica* transformations were conducted according to standard heat-shock protocols described in Sambrook and Russell (2001) and Barth and Gaillardin (1996). Total RNA isolation from *Y. lipolytica* cells, plasmid isolation from *E. coli*, DNA fragments’ extraction from agarose gel, and purification of DNA fragments were all conducted using appropriate kits from A&A Biotechnology (Gdynia, Poland) – Total RNA Midi, Plasmid Mini, Gel-Out or Clean-Up. Restriction digestion of DNA fragments was done using either *Not*I enzyme (Thermo Fisher Scientific, Waltham, USA) or *Bsa*I (New England Biolabs, Ipswich, USA). Routine colony PCR with *E. coli* biomass was run using *Taq* DNA polymerase (A&A Biotechnology), while colony PCR with *Y. lipolytica* biomass was conducted using Phire Hot Start II DNA Polymerase (Thermo Fisher Scientific). All the reactions and protocols were conducted according to the manufacturers’ recommendations.

### Design of the double-gene constructs and Golden Gate reaction

**Fig. 1:**
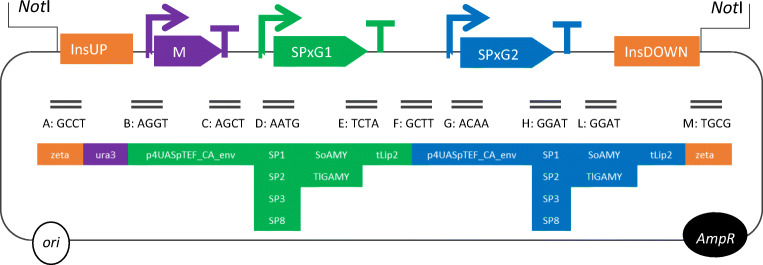
Design of double TU expression cassettes assembled via modular cloning. Schematic representation of the expression cassette variants. Modules are represented in color code: orange, insertion sites UP and DOWN (zeta); purple, complete gene encoding selection marker (M) *ura3* with truncated promoter and terminator; green (blue), first TU (G1 position) (second TU (G2)) composed of hybrid promoter (4 direct repetitions of UAS and a minimal promoter of pTEF with CA environment), four modules for SPs (SP1, SP2, SP3, SP8), two modules for gene encoding mature polypeptide (SoAMY, TlGAMY) and terminator (tLip2). Assembly scars are indicated as =, and corresponding 4 nt overhang sequences are given. Circular objects indicate *ori* of replication (white) and ampicillin resistance gene (black) contained in the bacterial part of the assembly, discarded prior to *Y. lipolytica* transformation through *Not*I endonuclease digestion (sites indicated)

Nucleotide sequences of the four signal peptides (SPs; SP1, SP2, SP3, SP8; given in Tab.S3) and of two heterologous genes encoding alpha-amylase *SoAMY* and glucoamylase *TlGAMY* were adjusted to a Golden Gate modular cloning system for *Y. lipolytica* as described previously (Celińska et al. 2017b; Celińska et al. 2018). The previously developed Golden Gate scaffold was narrowed to a double TU-bearing set of 4 nt overhangs, matching the corresponding destination vector pSB1A3-RFP, available from iGEM collection (http://parts.igem.org/Collections). The target genes were arranged in the double TU assemblies, differing in the type of SP transcriptionally fused to the heterologous genes, and the order of TUs bearing either *SoAMY* or *TlGAMY* gene (Fig. 1). The order of TUs is abbreviated in the text as G1XG2X, where G1 is the first TU position, G2 is the second TU position, and X is either S – *SoAMY* or T- *TlGAMY* (e.g., G1SG2T – *SoAMY* gene is located in the first TU – G1, and *TlGAMY* gene is located in the second TU – G2). In silico analyses of the target fragments (GGFs – Golden Gate Fragments) and in silico assembly were done using Benchling (https://benchling.com/) and confirmed with control restriction digestions. Construction of the Golden Gate assemblies (GGAs) followed previously described pipeline (Celińska et al. 2018). Briefly, pCR Blunt II TOPO vector (Thermo Fisher Scientific) and pSB1A3-RFP (iGEM collection) were used as the donor and destination vectors, respectively. Golden Gate reaction mixtures were composed of equimolar amounts of each GGF and the destination vector, T4 DNA ligase buffer, *Bsa*I restriction endonuclease, and T4 ligase (all from NEB). *E. coli* JM109 was transformed with the reaction mixture. Positive clones were isolated and expression cassettes were verified through sequencing (Genomed, Warsaw, Poland). Correct GGAs were linearized with *Not*I endonuclease and used for transformation of *Y. lipolytica* Po1h cells. Clones appearing after 48-h incubation at 30 °C on YNB-selection plates were replica-plated on fresh YNB, YPD, and YPS agar plates (g/L 10 yeast extract; 20, bacto peptone; 20, glucose; 10, starch; 15, agar). All the clones were screened for the presence of GGA expression cassettes through colony PCR, and its functionality was tested via starch-iodine drop test, as described previously (Celińska et al. 2016). Briefly, after 48 h culturing on YPS, the biomass was scraped and 5% iodine solution (I2 in KI) was poured onto the plate to visualize the translucent zones, indicating starch hydrolysis. All the strains bearing the expression cassettes (GGAs) and generating translucent zones in the starch-iodine drop test were deposited as glycerol stocks at − 80 °C.

### Gene expression analysis through RTqPCR in growth-phase synchronized cultures

Expression of *SoAMY* and *TlGAMY* genes was analyzed in growth-phase synchronized cultures of *Y. lipolytica* strains bearing operable expression cassettes (one of the eight variants generated). Each representative strain was cultured in independent duplicate and subjected to separate RNA isolation. Growth-phase synchronization was conducted according to Ruiz-Herrera and Sentandreu (2002) with modifications. Strains were cultivated in YPD medium (5 mL in 15 mL test tube) at 30 °C, 250 rpm, over 23 h. The pre-cultures were then centrifuged (4000 rpm, 4 °C in Eppendorf 5430 R centrifuge; Eppendorf, Hamburg, Germany); the biomass was resuspended in 5 mL of sterile ddH2O (4 °C) and incubated for 2 h at 4 °C (inverted occasionally). Subsequently, 1 mL of the synchronized pre-cultures was inoculated into 30 mL of YPD medium in 100-mL Erlenmeyer flask, and the cultures were continued at 30 °C, 250 rpm, over 23 h. Afterwards, the biomass from 10 mL of the cultures was used for isolation of total RNA. Quantity and integrity of isolated RNA was verified through gel electrophoresis and spectrophotometric measurement (NanoDrop; Thermo Fisher Scientific). RNA was then transcribed into cDNA using SuperScript III Reverse Transcriptase and oligo(dT) primer, according to the manufacturer’s protocol (Thermo Fisher Scientific). Obtained cDNA preparations were used as templates in RTqPCR, carried out in an Applied Biosystems 7500 device (Applied Biosystems, Foster City, USA). Primers for real-time qPCR were designed with Primer Expert Software (Applied Biosystems) and are listed in Tab.S3. The reactions were set up using RT HS-PCR Mix SYBR® B (A&A Biotechnology) in total volume 25 μL, according to the manufacturer’s protocol. LoROX dye was used as a passive reference. Primers were analyzed for their amplification efficiency by running RTqPCR reaction on a series of twofold-diluted template. The following thermal profile was adopted: 95 °C 4 min, (95 °C 15 s, 62 °C 15 s, 72 °C 30 s) × 40, 72 °C 1 min, Melt Curve 94 °C 15 s, 60 °C 60 s, 95 °C 30 s, 60 °C 15 s. Fluorescence from SYBR®Green was measured at the elongation step. Samples were analyzed in triplicate. The obtained data were processed according to ΔCt method (Livak and Schmittgen 2001), enabling estimation of overall expression level of *SoAMY* and *TlGAMY* genes in individual strains in relation to *ACT1* (presumed to be a house-keeping gene having stable expression level).

### Small-scale cultivations – quantitative evaluation of amylolytic phenotype in different starch types

*Y. lipolytica* recombinant strains bearing one of the eight GGAs were subjected to quantitative phenotype examination in liquid cultures with starch as the main carbon source. First, five sub-clones representing specific cassette construction were subjected to pre-screening for acquired amylolytic activity by cultivations on cooked starch (according to methodology described in the next section). The reference, prototrophic Po1h strain was each time cultured simultaneously. Subsequently, three sub-clones with negligible variability in the analyzed trait were cultured in media containing one of three starch types: rice (Sigma-Aldrich; Merck KGaA, Saint Louis, USA), corn (Sigma-Aldrich), and potato, soluble (POCh; Avantor Performance Materials Poland, Gliwice, Poland). The cultivations were conducted in either raw or cooked starch. Due to technical limitations concerning mixing of raw starch, the cultivations with raw and liquefied substrates were conducted in different vessels and volumes (described in detail in the two following sections).

### Cultivations on cooked starch

Selected recombinant strains were spread on YNB agar medium and incubated at 30 °C for 24 h. Liquid pre-cultures were developed in 200 μL of YPD medium in MTP plates, incubated in an MTP thermo-shaker (Biosan) at 30 °C, 150 rpm for 24 h. Subsequently, 5 μL of the pre-culture were transferred into 200 μL of production medium in MTP (g/L 5, starch; 2, glucose; 1, yeast extract; 2, bactopeptone in 0.1 M phosphate buffer Na-K, pH 5.7) and cultured over 72 h in 30 °C, 250 rpm (ES-20, Orbital Shaker-Incubator; Biosan). Each of the sub-clones and the reference strain were cultured in biological triplicate.

### Cultivations on raw starch

The strains were prepared analogously as mentioned in the preceding section; but for this experiment, the pre-cultures were conducted in 5 mL of YPD medium (in 15-mL test tubes). After 24 h in 30 °C, 250 rpm, the pre-cultures (150 μL) were transferred into 5 mL of the production medium. Composition of the production medium was identical as for cooked starch cultures with the difference that raw, non-liquefied starch was added directly prior to inoculation, and chloramphenicol was added for additional anti-microbial protection (up to 10 mg/L). Additionally, glass beads (3 mm in diameter) were added into the tubes, in order to improve dispersion of raw starch. The cultures were continued for 72 h, at 30 °C, 250 rpm (ES-20, Orbital Shaker-Incubator; Biosan). Each of the sub-clones and the reference strain were cultured in biological triplicate.

### Production cultures in flasks

Pre-cultures of selected strains (F215 and C185) were developed from colonies grown in YPD agar plate, inoculated to 50 mL YPD medium, cultivated for 22 h, at 30 °C, with shaking 250 rpm. Ten milliliters of the pre-culture were transferred into 1-L Erlenmeyer flasks, with medium composed as follows: (g/L) 40, starch; 10, yeast extract; 20, peptone in 0.1 M phosphate buffer Na-K, pH 5.7. The final culture volume was of 100 mL.

To ensure maximum starch hydrolysis in control cultures, external supplementation with enzymatic, amylolytic preparation was conducted. The preparation dose (the amount of amylolytic activity units per gram of starch in the medium) was established in a separate experiment conducted under the same conditions, in the same culture medium, but without yeast cells. Three doses were tested: 0, 2.5, 5, and 7.5 mL per 100 mL of culture, and based on observed kinetics of starch degradation, the dose 2.5 mL was chosen for the production cultures. Production cultures of F215 and C185 strains, with or without supplementation with amylolytic preparation, were conducted at 31 °C, with shaking 250 rpm, for 72 h with intermittent samples collection. Each culture variant was conducted in biological duplicate.

### Batch production cultures in bioreactors

Selected superior amylolytic strain F215 (SP3 G1TG2S) was first propagated in 50 mL YPD medium, at 30 °C, with shaking 250 rpm over 22 h. The pre-culture was then transferred into Infors 2 (Multifors) bioreactor of total volume 2 L, and culture medium volume 0.5 L. The culturing medium was as follows: yeast extract 10 g/L, peptone 20 g/L, rice starch 40 g/L. The C/N ratio of the medium was 8.23. Elementary composition of complex media constituents (yeast extract and peptone) was earlier determined through elementary analysis (Celińska et al. 2017a). The following conditions were maintained stable throughout the culturing time: temperature 31 °C, pH 5.5 by regulation with 40% NaOH and 10% H_2_SO_4_, oxygen saturation at 21% by setting cascade of mixing and total flow of compressed air.

Samples were collected periodically, centrifuged for 10 min at 15 krpm (Hareus) and stored at − 20 °C until analyzed. The supernatant was diluted and subjected to microSIT assay to determine starch hydrolysis progress, and HPLC analysis to determine concentration of citric acid, erythritol, and mannitol, according to protocol described previously (Kubiak et al. 2019). Yeast biomass accumulation was analyzed by a standard gravimetric methods, described previously (Celińska et al. 2017a).

### Analysis of starch hydrolysis degree – determination of residual starch concentration

The amount of residual starch contained in the post-culturing media was used as a measure of the recombinant strains’ amylolytic activity. The protocol for starch concentration assessment (microSIT) was described previously (Borkowska et al. 2019) with modifications regarding preparation of raw starch-based samples (described below). Each of the batch cultivations was analyzed in technical duplicate. The final results were expressed as a relative decrease in starch-iodine staining value in reference to its initial concentration. In calculations, the staining value of starch-iodine complexes that remained in the reaction mixture after digestion was subtracted from the staining value of the total starch-iodine complexes contained in the control samples (the substrate in the medium, acidified with 1 M HCl). Details on sensitivity and range of the analytical methods are given in the original report, where the micro-assays were described (Borkowska et al. 2019).

For the liquefied starch-containing samples, the cells were first separated from the post-culturing medium by centrifugation (4000 rpm, 10 min, 4 °C in Eppendorf 5430 R centrifuge; Eppendorf), and 40 μL of the resultant supernatants were transferred to a transparent flat-bottomed 96-well assay microplate (Corning, USA). The residual starch was stained by 50 μL of I2/KI solution (5 mM/5 mM) after acidification with 10 μL of 1 M HCl. The absorbance of the samples at 580 nm wavelength was analyzed using a Tecan Infinite M200 automatic plate reader (Tecan Group Ltd., Männedorf, Switzerland). The readouts obtained for the recombinant strains were normalized vs Po1h reference strain, and the results were presented as relative values with respect to the reference.

The raw starch-containing samples were processed correspondingly, with the difference that the starch granules were initially cooked prior to the test. Briefly, after through vortexing, 40 μL of the post-culturing liquid was transferred into 160 μL of phosphate buffer Na-K pH 5.7 in 96-well semi-skirted PCR plates (4-titude, UK) tightly covered with microplate sealing mats (Axymat, Axygen) and boiled for 60 min at 99.9 °C in a Verity 96-well Thermal Cycler (Applied Biosystems). Forty microliters of the boiled post-culturing medium were transferred to a transparent flat-bottomed 96-well assay microplate, and processed accordingly as the liquefied starch-containing samples. All the dilution factors were considered upon the final results calculations.

### Determination of lipid content and fatty acid profile in yeast cells

Quantification of lipids and the determination of FA profile were performed according to Browse et al. (1986). Briefly, biomass from 2-mL culture sample was freeze-dried and sealed under nitrogen. Methanolic HCl was used to digest the biomass and methylate FAs. The process was carried out in an atmosphere of nitrogen. Following digestion/methylation, FAMEs were extracted into hexane. The organic phase was then analyzed using a 7890A gas chromatograph (AgilentTechnologies, CA, USA) equipped with an S/SL inlet operated in split mode with a 50:1 split ratio. Injection volume was 1 μL. FAMEs were separated on a WAX plus column (25 m × 0.25 mm × 25 μm; Phenomenex, CA, USA). FID was used to detect the eluting analytes. Quantitation was based on the addition of 50 μg of C17:0 to each sample as an internal standard. Supelco 37 Component FAME Mix (Sigma-Aldrich, PA, USA) was used to identify the peaks.

### Statistical analysis

Statistical importance of the differences between compared sets of data was analyzed using one-way analysis of variance (ANOVA) and Tukey’s multiple comparison tests. Distributional assumptions for applying ANOVA analyses were assessed by the Shapiro-Wilk test, while homogeneity of variances between the subjects was assessed using Levene’s tests. Statistical analyses were performed with the STATISTICA data analysis software system (StatSoft, Inc., Tulsa, OK, USA). The results were considered to be statistically different at a *p* value of 0.05 or less. The results were expressed as mean ± standard deviation (± SD) of the replicates, as indicated above. Graphical presentation of the obtained data was done using the Microsoft Excel 2013 software.

## Results

### Design and construction of *Y. lipolytica* recombinant strains

Design of the double TU-bearing DNA constructs followed previously developed protocols (Celińska et al. 2017b; Celińska et al. 2018) with modifications. In this study, individual transcription units, bearing either *SoAMY* or *TlGAMY* gene fused with SP (SP1, SP2, SP3, SP8) and flanked with regulatory elements (p4UASpTEF promoter, tLip2 terminator), were located interchangeably in the first or the second position of the expression cassette (termed G1 or G2). Auxotrophy selection marker-encoding gene (*ura3d1*) and genomic integration elements (*zeta*) remained unchanged amongst the variants (see Fig. 1). Selection of SPs was based on our previous findings, where 10 different SPs were fused to 2 reporter polypeptides—SoAMY and TlGAMY (as in the present study) (Celińska et al. 2018). Previously, the genes were cloned individually in *Y. lipolytica* strains, to evaluate their specific secretory efficiency for the two polypeptides. It was possible to indicate more/less robust secretory tags having corresponding effect on the two tested polypeptides. The most efficient SPs, namely, SP1 (spYALI0B03564g; similar to 1,3-glucosidase precursor), SP2 (spYALI0D20680g; cell wall protein with similarity to glucanases), SP3 (spYALI0E22374g; similar to GPI-anchored aspartyl protease 3), and SP8 (insect alpha-1,4-glucan-4-glucanohydrolase; here referred to as SoAMY), were used in this study.

**Fig. 2:**
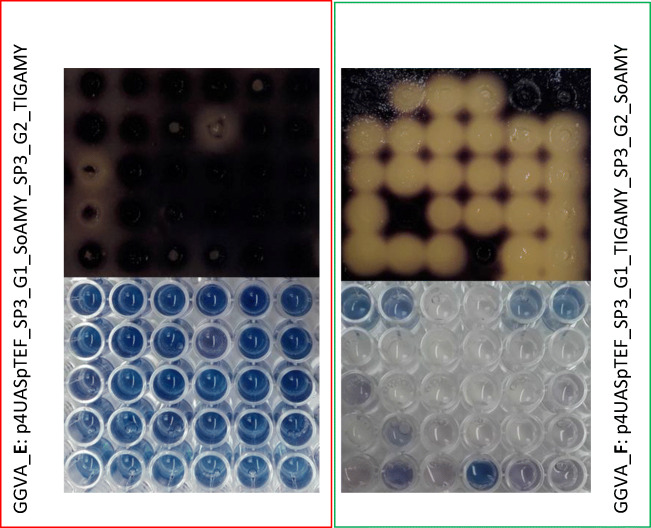
Prevalence of a specific amylolytic phenotype of *Y. lipolytica* strains acquired via transformation with expression cassettes GGA E (framed in red) and GGA F (framed in green) assayed in drop test on agar YPS plate (top panel) and in microSIT assay (bottom panel). Top panel: YPS plate stained with iodine after growth of different recombinant *Y. lipolytica* strains (iodine drop test); separate strains were spread using stainless steel replicator; translucent zones indicate degree of starch consumption by the strains. Bottom panel: Stained microSIT reactions conducted according to Borkowska et al. (2019); the more intensive blue color, the more starch remained undigested

Recombinant strains confirmed to bear heterologous genes by colony PCR and demonstrating amylolytic phenotype (by iodine drop test; Fig. 2) were subjected to quantitative evaluation of phenotype in liquid cultures. Several transformants, representing each DNA constructions, were analyzed to ensure that inter-clone variation is negligible (as demonstrated in Park et al. 2019; Theron et al. 2020).

### Growth of recombinant strains on starch of different plant origin (rice, corn, potato) and types (raw, cooked)

The obtained recombinant strains were tested with respect to their acquired amylolytic activity towards starch of different plant origin, in either raw or cooked form. The experimental design initially covered 6 types of starch: cooked rice (CR), cooked corn (CC), cooked potato (CP), raw rice (RR), raw corn (RC), and raw potato (RP); however, due to technical-analytical problems, raw corn (RC) had to be eliminated from the final data analyses. Irrespective of the boiling time applied to that starch type prior to residual starch staining, it was impossible to gain clear, uniformly dispersed solution, which was required for spectrophotometric reads.

**Fig. 3:**
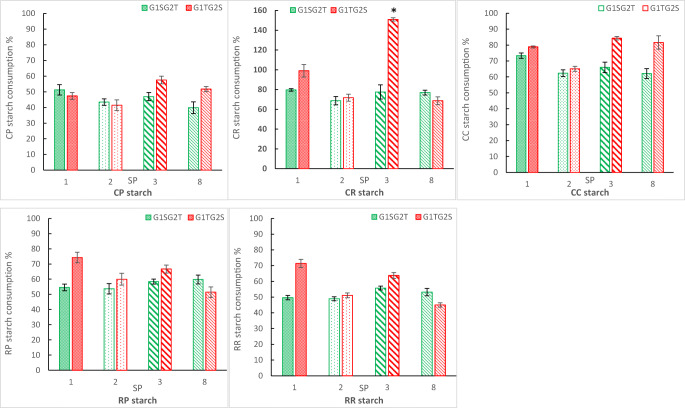
Starch hydrolyzing activity of *Y. lipolytica* recombinant strains bearing different variants of expression cassettes design (G1SG2T, G1TG2S) with the genes initiated with different 5′ sequences for SP (SP1, SP2, SP3, SP8) determined vs different types of substrates (CC, CP, CR, RP, RR). *X* axis, type of SP; *Y* axis, percentage value expressing hydrolysis degree of the indicated starch type vs negative control sample (%). Error bars indicate ± SE of replicates—three independent batch cultivations of three representative sub-clones, each sample analyzed in two technical replicates. *values > 100% result from design of the assay, i.e., its linearity range and normalization vs parental Po1h strain

The degree of the different starch type hydrolysis by sub-clones representing different expression cassette designs is shown in (Fig. 3). Primarily, we observed that transformation of *Y. lipolytica* host strain with SoAMY-TlGAMY-bearing cassettes conferred it with the ability to grow in starch as the main carbon source. Even more pronounced was a fact that the resultant strains could grow and utilize raw, non-pretreated starch of different plant origin. Considering quantitative evaluation of starch hydrolysis, the obtained data suggest predominance of G1TG2S assembly (*TlGAMY* gene in G1 position, *SoAMY* gene in G2 position) in providing more robust amylolytic phenotype (impact of the TU order on starch hydrolysis was statistically significant at *p* < 0.05) (Fig. 3; FIG.S1). Strains bearing G1TG2S-type of construction were generally more efficient in hydrolysis of starch, but the level of their predominance was different, depending on both—type of SP and the substrate. The least clear effect was observed for CP starch (*p* = 0.037), while the most obvious impact of the TU order was observed in CR, CC-based assays (*p* = 0.000). Furthermore, statistically important contribution of the SP type on the acquired amylolytic activity was observed (*p* < 0.05). The transformants in which the hydrolases were targeted for secretion by SP1 and SP3 performed better, while SP2-/SP8-bearing variants were less efficient. This trend was more or less clear, depending on the type of starch used in the cultures (Fig. 3). Finally, we observed that the strains bearing a cassette variant G1TG2S with SP3-targeted polypeptides were uniformly most efficient towards all starches in cooked form. Strains bearing the same order of TUs, but with the polypeptides targeted for secretion by SP1, were particularly efficient on raw starches. The strains bearing G1SG2T with SP8-targeted polypeptides represent the only example, where this cassette design triggered more efficient amylolytic phenotype than the opposite organization of TUs in two types of starchy substrate (RR, *p* = 0.019; CR, *p* < 0.05; RP, only tendency at *p* > 0.05). Irrespective of the cassette design or the starch type, transformants bearing polypeptides targeted for secretion by SP2 operated poorly, and no statistically important differences between the cassette designs were observed, which is unique amongst tested variants.

### Expression level of heterologous genes located in TU1 and TU2

**Fig. 4:**
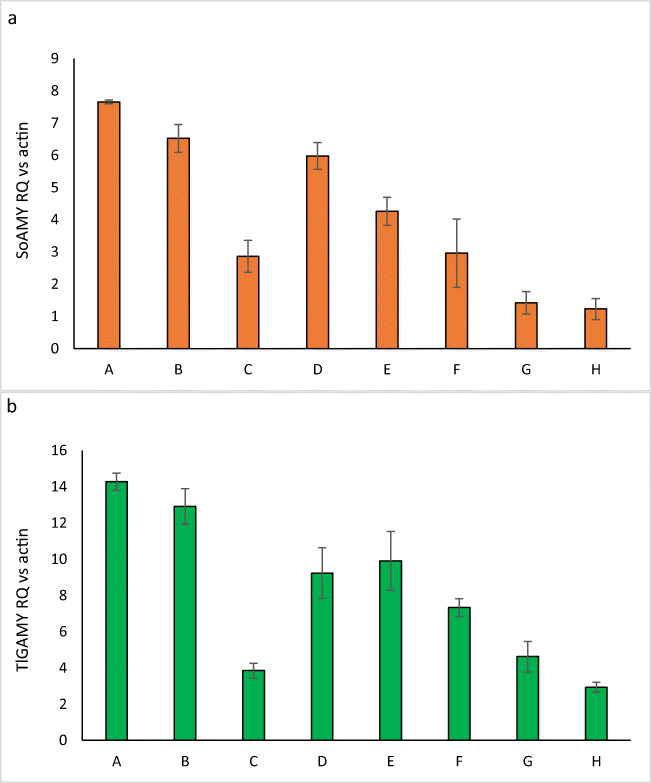
*SoAMY* and *TlGAMY* genes’ expression analysis vs actin in *Y. lipolytica* recombinant strains’ synchronized cultures, bearing different cassettes designs. *X* axis, strains variants A – SP1 G1SG2T, B – SP1 G1TG2S, C – SP2 G1SG2T, D – SP2 G1TG2S, E – SP3 G1SG2T, F – SP3 G1TG2S, G – SP8 G1SG2T, H – SP8 G1TG2S. *Y* axis, relative quantitation value normalized to that of actin, calculated according to a ΔCt method. Error bars indicate ± SD of cDNA relative quantitation

To get a deeper insight into the background behind variability amongst the obtained *Y. lipolytica* variants, we analyzed expression level of the genes cloned in the first and the second TU (G1 or G2). The strains subjected to this analysis were confirmed to bear an estimated single copy of the heterologous expression cassette, using RTqPCR on genomic DNA (FIG.S2.A), as described previously (Celińska et al. 2016). Gene expression analysis was conducted in growth-phase synchronized cultures to minimize variability of the analysis (Ruiz-Herrera and Sentandreu 2002). The results of *SoAMY* and *TlGAMY* genes’ expression level vs actin-encoding gene are presented in Fig. 4. The correlation coefficient between relative quantitation values for *SoAMY* and *TlGAMY* expression was relatively high (*r* = 0.7543). Conducted statistical analysis revealed that the 5′ sequence of the expressed hybrid genes (SP-encoding region) had a significant impact on the observed expression pattern (*p* < 0.00001), while positioning of a given gene in TU1 or TU2 is a not an important variable for gene expression level (*p* = 0.127) (graphs representing statistical analysis results are shown in FIG.S2.B).

### Lipids production from starch in flask cultures – comparison of extreme phenotypes

**Fig. 5:**
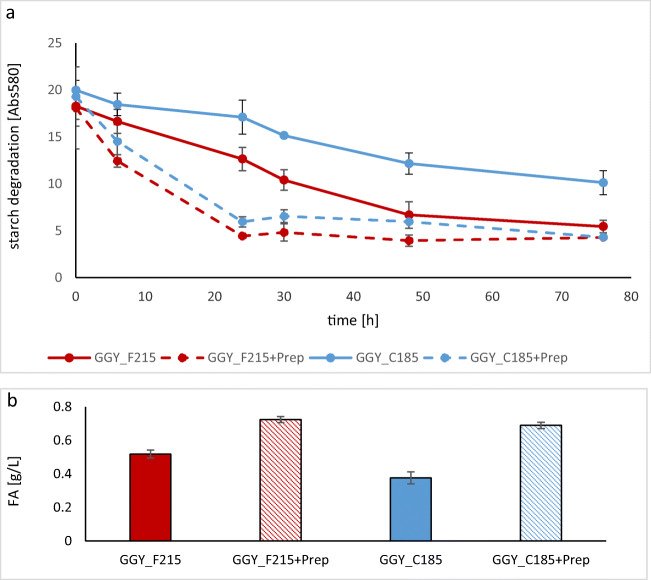
Comparison of starch hydrolysis (**a**) and fatty acids accumulation (**b**) by two amylolytic *Y. lipolytica* strains F215 and C185 in flask production cultures, with and without external supplementation with amylolytic preparation. **a***X* axis, culturing time (h); *Y* axis, starch concentration determined by iodine staining and absorbance measurement at 580 nm (%Abs580). **b***X* axis, type of strains and culture (with/without enzymatic preparation, “Prep”); *Y* axis, amount of FA contained in the yeast biomass, expressed in (g/L). Error bars indicate ± SD from two independent cultures, each analyzed in technical duplicate

To evaluate contribution of expression cassette optimization on the engineered strains’ biotechnological performance, their lipid production capacity was compared. To this end, representative strains showing the best and the worst acquired starch hydrolyzing activity (SP3-G1TG2S (GGY_F215) and (GGY_C185) SP2-G1SG2T; Tab.S1) were subjected to flask cultivations with starch as the main carbon source. Additionally, controls with external supplementation with alpha-amylase and glucoamylase were conducted, for maximum starch degradation rate. Dose of the preparation was pre-determined, and the results are shown in FIG.S3.A.B. Starch hydrolysis and lipids production in supplemented and non-supplemented flask cultures of F215 and C185 strains are presented in Fig. 5a and b. Expectedly, upon external supplementation with amylolytic enzymes, starch hydrolysis rate and lipids production were not significantly different between F215 and C185 strains (0.72 ± 0.018 vs 0.69 ± 0.019 FA [g/L] ± SD; *p* < 0.05), but were importantly different in non-supplemented cultures (0.52 ± 0.024 vs 0.37 ± 0.036 FA [g/L] ± SD for F215 and C185, respectively; *p* < 0.05). Comparison of cultures supplemented and non-supplemented with amylolytic preparation demonstrates that (1) the adopted strategy of strain optimization brought improvement in the analyzed trait—amylolytic activity, as the optimized strain (F215) was closer to maximum starch hydrolysis rate, ensured by external enzymes in the control cultures; (2) in the non-supplemented cultures, the strain selected based on its enhanced amylolytic activity (F215) was characterized by better growth and higher lipids accumulation, than the inferior variant (C185); and (3) under supplementation with external enzymes, the strains grew and accumulated comparable amounts of lipids, indicating that the only reason for better lipids accumulation by F215 is higher provision of nutrients in the non-supplemented cultures.

### Bioreactor cultures of the optimized biocatalyst with starch as the sole carbon source

**Fig. 6:**
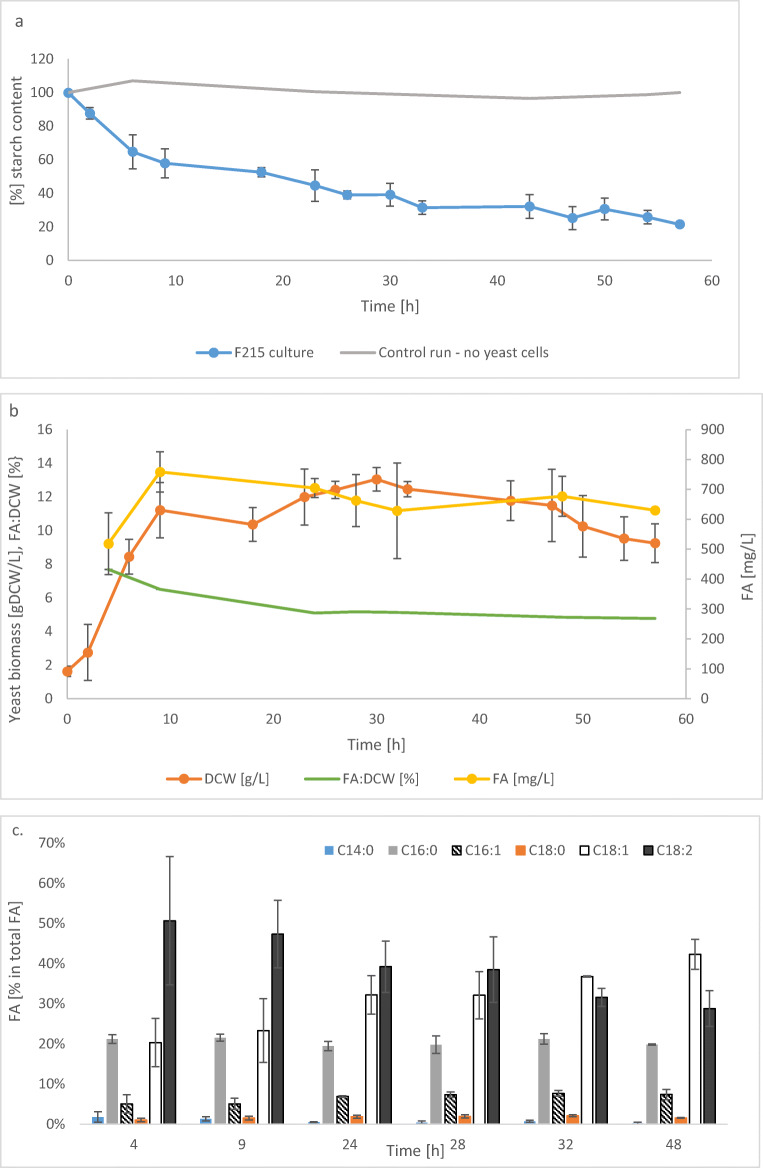
Bioreactor batch cultivation of optimized *Y. lipolytica* strain F215 grown on non-pretreated starch. Starch content vs starting amount (%) (**a**), biomass accumulation (gDCW/L) and total lipid content (% gDCW) or (mg/L) (**b**), and FA profile (%) (**c**). Control run – conducted without yeast cells, to asses degree of spontaneous starch degradation. *X* axis represents culturing time (h). *Y* axis represents percentage of total starch content vs its initial amount (**a**), grams of dry cellular weight, milligrams of FA per liter of culture (**b**; main axis), percentage content of FA in DCW (**b**; side axis), percentage value of a respective FA in total FA (**c**). Color code is explained in the legends. ± SD represents standard deviation out of four independent bioreactor culture runs

Finally, the most efficient amylolytic strain (F215) was subjected to bioreactor cultivations with starch as the sole carbon source. Averaged kinetics from four independent runs is presented in Fig. 6. To allow for possible spontaneous starch degradation due to prolonged mixing and heating, a control run, without yeast strain, was conducted. As presented in Fig. 6a, starch was gradually hydrolyzed up to approximately 60 h of culturing (78.5 ± 1.5% at 60 h). The most rapid hydrolysis of starch was observed within the first 48 h of culturing (up to 74.73 ± 6.83% at 48 h) which was also reflected by biomass growth (peak at 30 h of 13.04 ± 0.69 [gDCW/L] (Fig. 6b)). After that time, metabolic activity of the strain ceased, which resulted from exhaustion of nutrients, as illustrated by decreasing amount of lipids accumulated within the cells (from 7.69 ± 0.004 to 4.85 ± 0.001 %FA:DCW) (Fig. 6b). The highest fraction of total FA was represented by either C18:0 or C18:1, and their relative ratio changed over the culturing time (Fig. 6c). On the other hand, the relative content of C16:0 and C16:1 remained relatively unchanged (approx. 20% and 7%) throughout the culturing time. Key metabolites production (erythritol, mannitol, and citric acid) was negligible in these cultures (ERY, 0.21 ± 0.036; MAN, 0.7 ± 0.093; CA, 0.44 ± 0.056 [g/L] ± SD).

## Discussion

In this study, we aimed at optimization of starch-utilizing *Y. lipolytica* strains through manipulation with multi-gene construction design in terms of SPs and TU order. Consequently to our previous studies, we used amylolytic activities and starchy substrates as an easy to follow model, conferring strains with consolidated biocatalyst characteristics. Nevertheless, it has been recently pointed that starch-rich waste and by-product streams generated by bakery, confectionery, and wheat-milling plants could be employed as the sole raw materials for generic cultivation media suitable for microorganisms (Tsakona et al. 2014; Tsakona et al. 2019). Such food waste streams emerge as a potential feedstock for the synthesis of microbial bioproducts, including lipids, in the frame of the circular economy concept. Within that concept, optimization of consolidated biocatalysts able to grow in starchy substrate-based, complex media gains significant importance.

For reliable comparison of different recombinant strains, it is necessary to analyze several sub-clones and to assure minimum variability in the parameters that are not a subject of the analysis. Any changes in synthetic DNA construction can be easily and precisely monitored, while integration site within the host genome is more difficult to direct, especially in the case of host that show preference towards NHEJ mechanisms, as *Y. lipolytica* (Kretzschmar et al. 2013). It is accepted that integration of a recombinant DNA construction into less or more transcriptionally active site in the *Y. lipolytica* genome, or the number of copies integrated with the host genomic DNA, may impact expression level of transgenes and consequently resultant phenotype (Le Dall et al. 1994; Ogrydziak and Nicaud 2012). In the present study we used zeta flanking elements and nonspecific integration into Po1h zeta-less strain, which is a commonly accepted, reliable strategy (Pignede et al. 2000; Bordes et al. 2007). Recent studies in both *P. pastoris* and *Y. lipolytica* demonstrated reasonable neutrality between different integration loci from amongst the commonly used integration targets (Vogl et al. 2018; Holkenbrink et al. 2018). It has been revealed that nonspecifically integrated transformants showed highly uniform expression that was comparable with specific integration, suggesting that nonspecific integration does not negatively influence expression (Vogl et al. 2018). To minimize a risk of sub-clonal variation due to the cassette copy number or integration site, after transformation, five positive transformants bearing each type of construct were pre-screened for acquired amylolytic activity. Out of the pre-screened pool, three representative strains demonstrating negligible differences in starch consumption rate were subjected to further analyses. Such pre-selection strategy has been recently successfully used (Theron et al. 2020) and demonstrated that the following inter-clone variation was negligible.

Considering fundamental output of here conducted genetic manipulation, the obtained recombinant strains were conferred with the ability to grow on starch as the main carbon source, either in pretreated or raw state. Secondly, the amount of substrate released from the biopolymer was sufficient to support growth of the recombinant strains—each time higher than the reference strains, but varying, depending on the substrate and the recombinant strain variant (Fig. 3). It is well known that starches of different plant origin are highly variable in terms of their susceptibility to degradation, depending on their origin and characteristics of a given enzymatic activity (Baker 1992; Baker and Woo 1992; Sarikaya et al. 2000). Correspondingly, in our previous study, we demonstrated that depending on the exploited starch type, amylolytic effect exerted by the recombinant SoAMY alpha-amylase differed dramatically (Celińska et al. 2015a). In the only previous study on construction of *Y. lipolytica*-based consolidated biocatalyst able to utilize starch, the authors tested the obtained strains growth in either soluble starch, wheat raw starch, or industrial product containing starch (DZ starch; characteristics not provided) (Ledesma-Amaro et al. 2015). The different starchy substrates were used in different experiments (at different level of the biocatalysts testing), and the strains’ performance on those starch substrates was not systematically compared. The results presented in this study demonstrate how evaluation of a given strain’s biotechnological potential can differ, depending on the type of used starchy substrate. Furthermore, testing the obtained recombinant strains towards starch of different plant origin introduced variability to the results, reflected by both the overall level of the substrate degradation (the highest for CR, followed by CC, with the lowest values observed for CP) and the distribution of most/least efficient recombinants, representing genetic constructs differing in SPs and the positional order of TUs (Fig. 3). For example, strains bearing G1TG2S constructs with SP1 sequences were particularly efficient in consumption of raw starch (Fig. 3). Their predominance in raw starch digestion was clear when compared with the strains bearing the same order of TUs but different SP, and when compared with the strains bearing alternative organization of TUs and genes initiated with the same SP. Corresponding conclusions were withdrawn for the strains bearing the genes initiated with SP3 sequences. With respect to cooked starch hydrolysis, SP3-equipped G1TG2S variant turned out to uniformly endow the resultant strains with efficient amylolytic phenotype. While in the case of raw starch, the degree of the substrate hydrolysis was rather comparable (RR vs RP); cooked starch of different plant origin showed highly variable susceptibility towards SoAMY and TlGAMY action, ultimately leading to variable level of the substrate consumption. Integration of the results on gene expression (Fig. 4) and starch hydrolysis (Fig. 3) shows that genes fused at 5′ terminus with sequences encoding SP1 were expressed at higher level, which could contribute to the observed efficient amylolytic phenotype. However, no direct, straightforward relationship between a specific gene localization (G1 or G2), its expression level, and the overall amylolytic activity could be observed. It suggests that differences in the ability to hydrolyze starch between G1SG2T and G1TG2S strains do not derive directly from differences in the gene expression level. It is commonly accepted that the final enzymatic activity is not a first-order function of a given recombinant gene’s expression level, as evidenced earlier, also for *Y. lipolytica* (Dulermo et al. 2017).

In the only previous literature report describing starch-utilizing *Y. lipolytica* (Ledesma-Amaro et al. 2015), the obtained strain was tested for lipids production. In that study, to maximize production of FA, the C/N ratio of the culturing medium was set at 60 and 90, resulting in enhanced FA accumulation from 4.4 to 7.2 FA %DCW (0.49 vs 0.8 total FA g/L). Even more pronounced effect was observed when the two “amylolytic” genes were expressed in “a lipid overproducer” strain, heavily modified in FA and TAG turnover net (Beopoulos et al. 2008; Dulermo and Nicaud 2011; Beopoulos et al. 2012; Dulermo et al. 2013). In such background, lipids accumulation increased to 21.1 and 27% (FA %DCW; 2.44 ± 0.15 and 3.34 ± 0.13 g/L, for C/N ratio 60 and 90, respectively). In the present study, the microbial lipid accumulation in bioreactor culture (Fig. 6b) was at a level comparable with previous reports for a strain not engineered with respect to TAG turnover cultivated on carbohydrates (0.64 ± 0.08 g/L of lipids, 7.69–4.85% DCW, depending on the culturing time), even though the C/N ratio of the present medium equaled to 8.23 (not optimal for FA accumulation, but promoting growth and enzymes synthesis). Nevertheless, to generate highly efficient lipid producer from starchy wastes, the optimized cassette could be transformed into a lipid overproducer strain background, as previously (Ledesma-Amaro et al. 2015). Strains optimized in this trait, upon cultivation in a medium of high C/N ratio, can accumulate as much as 30–70% of total lipids (Beopoulos et al. 2008; Tai and Stephanopoulos 2013; Blazeck et al. 2014). On the other hand, we observed that here obtained, optimized strain (F215) exhibited much faster substrate hydrolysis in time than the starch-utilizing *Y. lipolytica* strain constructed previously (Ledesma-Amaro et al. 2015). In that previous study, starch hydrolysis ranged between 49.4 ± 2.4% to 60.3 ± 6.4% (29.64 to 54.27 g/L in 168 h, depending of the strain and culture medium) giving the total substrate degradation in time of 0.1769 and 0.323 g/(L*h). In the present study, substrate hydrolysis in time reached 0.64 and 0.4 g/(L*h) at the 48 h of culturing, with substrate hydrolysis rate ΔS/Δt ranging 2.45–0.14 g/(L*h) (Fig.S4).

Finally, here observed FA profile was corresponding to previous reports on FA profile in *Y. lipolytica* cultivated on standard substrates, like glycerol and glucose (Celińska and Grajek 2013), or more complex media like sugar beet molasses (Gajdoš et al. 2015), with dominance of unsaturated C18:2 and C18:1, followed by saturated form of C16:0. In previous study on cultivation of engineered *Y. lipolytica* on starch (Ledesma-Amaro et al. 2015), FA profile was mainly represented by C18:1, but the percentage contribution of C18:2 was much lower than in the present study. On the other hand, in that previous report, longer chain FA (C24:0) was produced at detectable level, which was not the case in the current study. This difference could result from differences in technical/analytical approach. Interestingly, the observed FA profile, especially in terms of percentage content of C18:1 and C16:0, so the dominant FA detected, was also similar to FA profile observed in the other oleaginous yeast *Rhodosporidium toruloides* grown on cassava starch hydrolysate (Wang et al. 2012). The other compounds, typical for *Y. lipolytica* metabolism, were detected at surprisingly low levels. Erythritol, mannitol, and citric acid were produced at the levels below 1 g/L. Importantly, the same observation was done in Ledesma-Amaro et al. (2015), where small molecular metabolites were detected at close to zero level.

Based on the current research, we conclude that apart from manipulation with promoter strength or the number of heterologous genes copies, the positional order of TUs and type of SPs targeting hydrolases for secretion could be considered as additional factors to be evaluated upon consolidated biocatalysts optimization. As evidenced, in this particular study, G1TG2S cassette design was generally more beneficial for the exerted amylolytic phenotype. Furthermore, usage of SP1 and SP3 secretory tags for targeting the hydrolyses to the extracellular space had a significant, positive impact on the acquired amylolytic activity. In the case of SP1-bearing fusion polypeptides, efficient amylolytic phenotype was associated with enhanced genes expression level. We systematically tested the constructed biocatalysts in different types of starches, showing high variability in their susceptibility to digestion by strains representing different variants of the cassette design. Finally, we observed that the conducted modifications conferred the host strains with optimized consolidated biocatalyst characteristics able to produce lipids from raw starch.

## Electronic supplementary material

ESM 1 (PDF 501 kb)
