# Supplementary material for: Optimization of Yarrowia lipolytica-based consolidated biocatalyst through synthetic biology approach: transcription units and signal peptides shuffling
Source: Appl Microbiol Biotechnol. 2020 May 2;104(13):5845–59. doi: 10.1007/s00253-020-10644-6 (PMC7306051; doi:10.1007/s00253-020-10644-6)

Applied Microbiology and Biotechnology

Optimization of *Yarrowia lipolytica*-based consolidated biocatalyst through synthetic biology approach: transcription units and signal peptides shuffling

Ewelina Celińska[*1], Monika Borkowska[1], Paulina Korpys-Woźniak[1], Monika Kubiak[1], Jean-Marc Nicaud[2], Piotr Kubiak[1], Maria Gorczyca[1], Wojciech Białas[1]

[1]Department of Biotechnology and Food Microbiology, Poznan University of Life Sciences, ul. Wojska Polskiego 48, 60-627 Poznań, Poland

[2]Micalis Institute, INRA-AgroParisTech, UMR1319, Team BIMLip: Integrative Metabolism of Microbial Lipids, Domaine de Vilvert, 78352 Jouy-en-Josas, France

*Corresponding author: Ewelina Celińska, e-mail: ewelina.celinska@up.poznan.pl, phone: +48618466007, ORCID 0000-0001-8372-8459

Table S1 *Yarrowia lipolytica* strains used in this study

Yarrowia lipolytica Po1h	*Genotype:*<br>*MATa, ura3–302, xpr2–322, axp1–2, pXPR2-SUC2*<br>derived from Po1f (ura3- leu2-)		Phenotype:<br>both extracellular proteases AEP, AXP deleted, Suc+, Ura−
The following strains are derivative of *Y. lipolytica* Po1h strain.<br>The strains were transformed with one of eight variants of expression cassette (GGA – Golden Gate Assembly).<br>Common elements of the GGAs are:<br>*InsUP:zeta_M:ura3_P1:p4UASpTEF_CAenv_SPx_G1_T1:tLip2_P2::p4UASpTEF_CAenv_SPx_G2_T2:tLip2_InsDOWN:zeta*<br>Variable elements are listed			
Strain No	GG Assembly	SP in G1 / SP in G2	Gene in G1, Gene in G2<br>S- SoAMY, T - TlGAMY
GGY174	GGA_A	SP1/SP1	G1SG2T
GGY175	GGA_A	SP1/SP1	G1SG2T
GGY176	GGA_A	SP1/SP1	G1SG2T
GGY177	GGA_B	SP1/SP1	G1TG2S
GGY178	GGA_B	SP1/SP1	G1TG2S
GGY180	GGA_B	SP1/SP1	G1TG2S
GGY185	GGA_C	SP2/SP2	G1SG2T
GGY187	GGA_C	SP2/SP2	G1SG2T
GGY188	GGA_C	SP2/SP2	G1SG2T
GGY189	GGA_D	SP2/SP2	G1TG2S
GGY190	GGA_D	SP2/SP2	G1TG2S
GGY191	GGA_D	SP2/SP2	G1TG2S
GGY194	GGA_E	SP3/SP3	G1SG2T
GGY195	GGA_E	SP3/SP3	G1SG2T
GGY196	GGA_E	SP3/SP3	G1SG2T
GGY206	GGA_F	SP3/SP3	G1TG2S
GGY207	GGA_F	SP3/SP3	G1TG2S
GGY215	GGA_F	SP3/SP3	G1TG2S
GGY217	GGA_G	SP8/SP8	G1SG2T
GGY219	GGA_G	SP8/SP8	G1SG2T
GGY220	GGA_G	SP8/SP8	G1SG2T
GGY221	GGA_H	SP8/SP8	G1TG2S
GGY222	GGA_H	SP8/SP8	G1TG2S
GGY225	GGA_H	SP8/SP8	G1TG2S

Table S2 *Escherichia coli* strains used in this study

Escherichia coli JM109	Genotype: *F'(traD36,proAB+,lacIq,Δ(lacZ)M15), endA1,recA1,hsdR17(rk-mk+), mcrA,supE44,λ, gyrA96,relA1, Δ(lacproAB),thi-1*			
The following strains are derivative of *Escherichia coli* JM109 laboratory strain.<br>The strains were transformed with indicated genetic construction, following previously set Golden Gate standard (Celinska et al., 2017)				
Strain No	GG item	Acquired resistance	Backbone vector	Insert characteristics
GGE 0115	GGV	KanR	TOPO	SP1 TlGAMY_G1
GGE 0116	GGV	KanR	TOPO	SP2 TlGAMY_G1
GGE 0117	GGV	KanR	TOPO	SP3 TlGAMY_G1
GGE 0118	GGV	KanR	TOPO	SP1 TlGAMY _G2
GGE 0119	GGV	KanR	TOPO	SP2 TlGAMY_G2
GGE 0120	GGV	KanR	TOPO	SP3 TlGAMY_G2
GGE 0100	GGV	KanR	TOPO	SP8 TlGAMY_G1
GGE 0101	GGV	KanR	TOPO	SP8 TlGAMY_G2
GGE 0087	GGV	KanR	TOPO	SP1_SoAMY_G1
GGE 0088	GGV	KanR	TOPO	SP2_SoAMY_G1
GGE 0089	GGV	KanR	TOPO	SP3_SoAMY_G1
GGE 0090	GGV	KanR	TOPO	SP4_SoAMY_G1
GGE 0091	GGV	KanR	TOPO	SP7_SoAMY_G1
GGE 0092	GGV	KanR	TOPO	SP8_SoAMY_G1
GGE 0093	GGV	KanR	TOPO	SP1_SoAMY_G2
GGE 0094	GGV	KanR	TOPO	SP2_SoAMY_G2
GGE 0095	GGV	KanR	TOPO	SP3_SoAMY_G2
GGE 0099	GGV	KanR	TOPO	SP8_SoAMY_G2
GGE 0106	GGV	KanR	TOPO	P2 p4UASpTEF
GGE 0108	GGV	KanR	TOPO	P1 p4UASpTEF
GGE 0037	GGV	KanR	TOPO	InsUP_zeta NotI
GGE 0038	GGV	KanR	TOPO	InsDOWN_zeta NotI
GGE 0021	GGV	KanR	TOPO	T2-3 Lip2
GGE 0014	GGV	KanR	TOPO	T1 Lip2
GGE 0013	GGV	KanR	TOPO	M ura3
GGE 0145	GGVA_A	AmpR	pSB1A3	p4UASpTEF_SP1_G1_SoAMY_SP1_G2_TIGAMY
GGE 0149	GGVA_B	AmpR	pSB1A3	p4UASpTEF_SP1_G1_TIGAMY_SP1_G2_SoAMY
GGE 0146	GGVA_C	AmpR	pSB1A3	p4UASpTEF_SP2_G1_SoAMY_SP2_G2_TIGAMY
GGE 0150	GGVA_D	AmpR	pSB1A3	p4UASpTEF_SP2_G1_TIGAMY_SP2_G2_SoAMY
GGE 0147	GGVA_E	AmpR	pSB1A3	p4UASpTEF_SP3_G1_SoAMY_SP3_G2_TIGAMY
GGE 0151	GGVA_F	AmpR	pSB1A3	p4UASpTEF_SP3_G1_TIGAMY_SP3_G2_SoAMY
GGE 0148	GGVA_G	AmpR	pSB1A3	p4UASpTEF_SP8_G1_SoAMY_SP8_G2_TIGAMY
GGE 0152	GGVA_H	AmpR	pSB1A3	p4UASpTEF_SP8_G1_TIGAMY_SP8_G2_SoAMY

Table S3 Oligonucleotides and longer synthetic DNA fragments used in this study

**Oligonucleotide / Sequence Name**	**DNA sequence 5' → 3'**
GGP_InsertUP_zeta_A_F_Not	GGTCTCTGCCTGCGGCCGCtgtcgggaaccgc
GGP_InsertUP_zeta_B_R	GGTCTCTACCTtctagcaaagtgctttgtgc
GGP_M_Ura3_B_F	GGTCTCTAGGTataacttcgtatagcatacattatacgaag
GGP_M_Ura3_C_R	GGTCTCTAGCTtcgcttcggataactcc
GGP_P1_4UAS_pTEF_hp4d_C_F	GGTCTCt AGCT CGATACGCGT
GGP_P1_4UASpTEF_D_R	GGTCTCtCATTCTTCGGGTGTGAGTTGAC
GGP_G1_SSP1_D_F	GGTCTCtAATGAAGTTCACATTTGCTGCCG
GGP_G1_SSP2_D_F	GGGGGTCTCtAATGAAGTTCTCCACCGCCC
GGP_G1_SSP3_D_F	GGTCTCtAATGAAATCTCTATTGCTGTCGC
GGP_G1_SSP8_D_F	GGTCTCtAATGAAGGTGCTCGCCCTGCTGG
GGP_G1_SoAMY_E_R	GGTCTCTTAGACTAGTGGTGGTGGTGG
GGP_G1_TlGAMY_E_R	GGTCTCTTAGATCAATGATGATGATGATGATG
GGP_T1_LIP2_E_F	GGTCTCTTCTAgtgtctgtggtatctaagctatttatc
GGP_T1_LIP2_F_R	GGTCTCTAAGCcgatttgtcttagaggaacgc
GGP_P2_4UASpTEF_GS_F_F	GGTCTCTGCTTGCATGCTGAGGTGTCTCACAAG
GGP_P2_4UASpTEF_GS_G_R	GGTCTCTTTGTCTTCGGGTGTGAGTTGACAAGG
GGP_G2_SSP1_G_F	GGTCTCTACAATGAAGTTCACATTTGCTGCCGTTACCG
GGP_G2_SSP2_G_F	GGTCTCTACAATGAAGTTCTCCACCGCCCTTCTGGC
GGP_G2_SSP3_G_F	GGTCTCTACAATGAAATCTCTATTGCTGTCGCTGCTGGCG
GGP_G2_SSP8_G_F	GGTCTCTACAATGAAGGTGCTCGCCCTGCTGG
GGP_G2_SoAMY_H_R	GGTCTCTATCCCTAGTGGTGGTGGTGG
GGP_G2_TlGAMY_H_R	GGTCTCTATCCTCAATGATGATGATGATGATGTCTC
GGP_T2_LIP2_H_F	GGTCTCTGGATgtgtctgtggtatctaagctatttatc
GGP_T3_LIP2_L_R	GGTCTCTACTCcgatttgtcttagaggaacgc
GGP_InsertDOWN_zeta_L_F	GGTCTCTGAGTcatgtgtaacactcgctctg
GGP_InsertDOWN_zeta_M_R_Not	GGTCTCTCGCAGCGGCCGCactgaagggctttgtgag
r-t SoAMY_614_F (RTqPCR)	CTACCGACCACGGATTTGCT
r-t SoAMY_688_R (RTqPCR)	AGGATCGGTAGAAGTGTCAATCACT
r-t_TlGAMY_488_F (RTqPCR)	TCATCTGGCCAATCGGTAGAA
r-t_TlGAMY_554_R (RTqPCR)	TCGAATGTAGTTTCGTTCCAATGT
r-t_actin_117_F (RTqPCR)	TCTCCCCAGCGACAAGTCA
r-t_actin_168_R (RTqPCR)	GGCCCGTGGCTTTGGT
TlGAMY without native SP<br>codon optimized	AGACCAGAAACAACCGTTCCTCACGCAACCGGTTCTTTGGATGATTTCTTGGCTGCA<br>CAATCACCAATCGCTTTCCAAGGTATTTTGAACAACATTGGTCCATCTGGTGCATAT<br>TCAGAAGGTGTTAATCCAGGTGTTGTTATTGCTTCACCATCTAAGCAAGATCCAGAT<br>TACTTCTACACTTGGGTTAGAGATGCTGCATTGACAGTTCAATATTTGGTTGAAGAA<br>TTGGTTGCTGGTAATGCTTCTTTGCAATTCTTGATCCAAGATTACATCTCTTCACAAG<br>CTAGATTGCAAACTGTTGAAAATCCATCAGGTTCTTTGTCTTCAGGTGGTTTAGGTG<br>AACCAAAGTTCCATGTTGATGAAACTGCTTTTACTGATTCTTGGGGTAGACCACAAA<br>GAGATGGTCCACCATTGAGAGCTATTGCAATGATCTCTTTCGCTAACTATTTGATCG<br>ATAACGGTCATCAATCAACAGTTGAAGATATCATCTGGCCAATCGGTAGAAACGAT<br>TTGTCATACGTTTCTCAACATTGGAACGAAACTACATTCGATATCTGGGAAGAAGTT<br>CATTCTTCATCTTTCTTTACTACAGCAGTTCAATATAGAGCTTTAGTTCAAGGTTCTG<br>CTTTGGCATCAAAGTTGGGTCATACTTGTGATAACTGTGGTTCTCAAGCACCACAAA<br>TTTTGTGTTTCTTGCAATCTTACTGGACTGGTTCACATATTTTGGCTAATACTGGTGG<br>TGGTAGATCTGGTAAAGATGTTTCAACTATTTTAGGTGTTATTGGTTCTTTCGATCC<br>AAATGCTGATTGTGATGATGTTACATTTCAACCATGTTCAGCTAGAGCATTAGCTAA<br>CCATAAGCAAGTTGTTGATTCTTTTAGATCTATCTATGCAATTAATGCTGGTATTCCA

	TCAGGTTCTGCAGTTGCTGTTGGTAGATATCCAGAAGATGTTTACCAAGGTGGTCA<br>TCCATGGTACTTGACTACAGCTGCAGCTGCAGAACAATTGTACGATGCAATCTATC<br>AATGGAACCATGTTGGTCATATCGATATCAACGCTGTTAATTTGGATTTCTTTAAAT<br>CTATCTATCCATCAGCTGCAGAAGGTACATACACTTCTGATTCATCTACTTTCCAAG<br>ATATCATCTCAGCAGTTAGAACATATGCTGATGGTTTCTTGTCTGTTATTGAAAAGT<br>ACACTCCACCAGATAATTTGTTGGCTGAACAATTCCATAGAGAAACAGGTATTCCAT<br>TGTCAGCTGCATCTTTAACTTGGTCTTATGCTGCATTGAATACAGCTGCACAAAGAA<br>GAGCTTCTATTGTTCCATCACCATGGAACTCAAACTCTACAGATTTGCCTGATAAGT<br>GTTCAGCAACTTCTGCTACAGGTCCATACGCAACACCAACTAATACAGCTTGGCCA<br>ACTACAACTCAACCACCAGAAAGACCAGCTTGTACTCCACCATCTGAAGTTACTTTG<br>ACTTTTAATGCATTGGTTGATACAGCTTTCGGTCAAAACATATATTTGGTTGGTTCT<br>ATTCCAGAATTAGGTTCATGGGACCCAGCAAATGCTTTGTTAATGTCAGCAAAATCT<br>TGGACTTCAGGTAATCCAGTTTGGACATTGTCAATTTCTTTACCAGCTGGTACTTCTT<br>TCGAATACAAGTTTATTAGAAAGGATGATGGTTCATCTGATGTTGTTTGGGAATCT<br>GATCCAAATAGATCATACAATGTTCCAAAAGATTGCGGTGCTAACACAGCTACAGT<br>AAACTCTTGGTGGAGACATCATCATCATCATCATTGA
SoAMY without native SP<br>codon optimized	CAGAAGGACCCCCACTTCCTAGATGGACGAAACACTATCGTTCACCTCTTCGAGTG<br>GAAGTGGGCCGACATTGCTTCCGAGTGTGAGAACTTCCTGTCGGTGAAGAACTTTG<br>CCGGTGTTCAGGTGTCTCCCCCTGCTGAGTCCGTGGTCGTTGAGGGCCGACCCTGG<br>TGGGAGAAGTACCAGCCTGTCTCCTACACCCTCAACAACCGAGGCGGAGACGAGG<br>CCGCTTTCTCCGATATGGTTTCGCGATGTAACAACGTGGGAATCCGAATTTACGTG<br>GACCTGGTCGCCAACCACATGGCTACTTCGAACGGCCAGGGATCTGCCGGTAACAC<br>CTGCGACCCCTCTTCCAAGTCTTACCCTGCTGTCTCGTACACTTCTGAGAACTTCCAT<br>ACCTCCTGTGACATCGATTACACTGACTCGTCTTCCATTCGAAACTGCGAGCTGACC<br>GGCCTCAAGGACCTGGATCAGTCCCAGGACTACGTGCGAGGAAAGATCGAGGAGT<br>ACATGAACCACCTCATTTCTCTGGGAGTGGCCGGTTTTCGAGTGGACGCCGCTAAG<br>CATATGTGGCCCGCCGACCTCCAGGCTATCTTCGGCTCCCTCAACGACCTGTCTACC<br>GACCACGGATTTGCTTCGGGTGCCCGAGCTTTCATCTTTCAGGAAGTGATTGACAC<br>TTCTACCGATCCTGTCAAGAACACTGAGTACACCGGTTTCGGCAAGGTGTGCGAGT<br>TCCTCTTTGGCAACGACCTGGGACCCGCCTTTCGAGGAGAGAACCCTCTCCATTACC<br>TGAAGAACTGGGGTACTGAGTGGGGCCTGCTCGACGGTGGCGATACCGTTTCGTT<br>CGTGGACAACCACGATAACGAGCGAGACTCTCAGATTTTCCTGCATTACACCAACG<br>ATAAGCCCTACAAGGCTGCCATGGCCTTCATGCTCGCTCACTCCTACGACACCACTA<br>CCCGAGTCCTGTCGTCTTACAAGTTTGACTCCTCGGATCAGGGTCCCCCTTCTAACG<br>GCGACGATATTCTGTCTCCCGAGTTCGGCTCCGACGGAGCCTGTACCAACGGCTGG<br>GTTTGCCAGCATCGATGGTCCCCTGTCTTCAACATGGTTGAGTTTCGAAACGTGGTC<br>TCGGGAACTGAGCTGACCAACTGGTGGGACAACGGTTCGCAGCAGATTGCCTTCTC<br>TCGAGGTGACAAGGGCTTTTACGCCGCTACTGTCAACGAGGATATCGCTACCTCTA<br>TTACTACCGGACTGCCCGACGGTTCCTACTGTGATGTCATGTCTGGCTCTCTGGTGA<br>ACGGCGCCTGCACCGGAAAGACTCTGACCGTGTCTGGCGGTCAGGTCTACGTTGA<br>GCTGGGTGGTGCTGAGCTGGAGGCTGCTGTCGCTATTCACGTCAACGCTAAGCTG<br>CACCACCACCACCACCACTAG
SP1 (YALI0B03564g)	ATGAAGTTCACATTTGCTGCCGTTACCGCCGCGCTGGCCTCGTCCGCCATTGCC
SP2 (YALI0D20680g)	ATGAAGTTCTCCACCGCCCTTCTGGCTCTGGCCGCCGTCGCCACTGCC
SP3 (YALI0E22374g)	ATGAAATCTCTATTGCTGTCGCTGCTGGCGGTCCCGGCCACTGCC
SP8 (SoAMY NATIVE)	ATGAAGGTGCTCGCCCTGCTGGTTACTGTCTGCTTTTCCGTTGCCTCTGCC
**Plasmids**	
**Plasmid Name**	**Reference**
pSB1A3-RFP	iGEM collection (http://parts.igem.org/Collections)
pCR Blunt II TOPO vectors	Thermo Fisher Scientific

Figure S1. Starch hydrolyzing activity of Y. lipolytica recombinant strains bearing different variants of expression cassettes design (G1SG2T, G1TG2S) with the genes initiated with different 5' sequences for SP (SP1, SP2, SP3, SP8) determined vs different types of substrates (CC, CP, CR, RP, RR). The obtained results were presented to visualize contribution of the TUs order solely to acquired amylolytic activity towards starch, to illustrate results of statistical analysis. The same data are presented in **Fig.3.** in the manuscript, but presented differently. X axis: expression cassette design; Y axis: percentage value expressing consumption of the indicated starch type versus negative control sample [%]. Error bars indicate ±SE of replicates - three independent batch cultivations and two technical replicates for strains bearing different SPs representing specified TU organization.

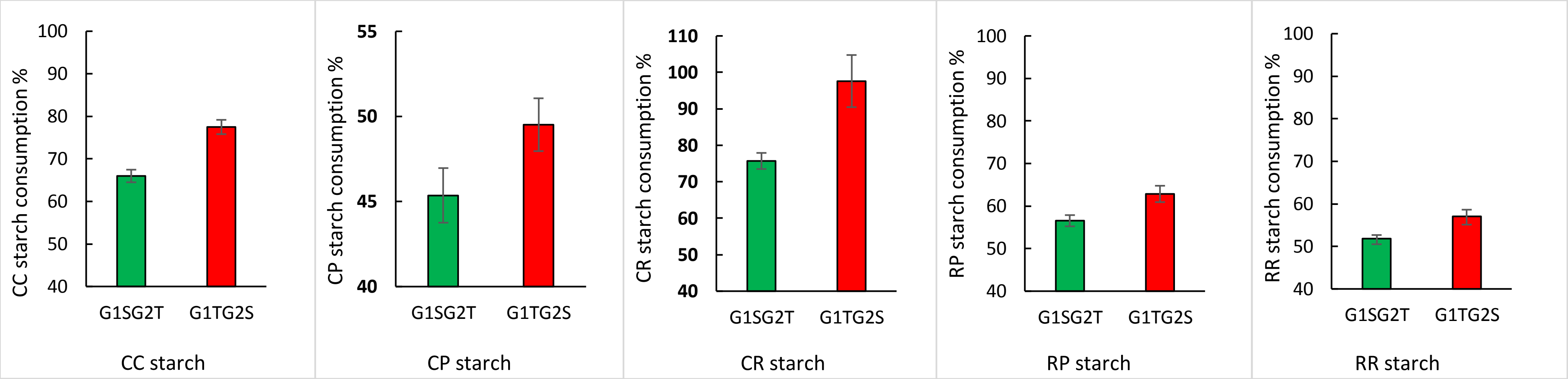

Figure S2

Fig.S2.A. Estimation of expression cassette copy number based on RTqPCR analysis

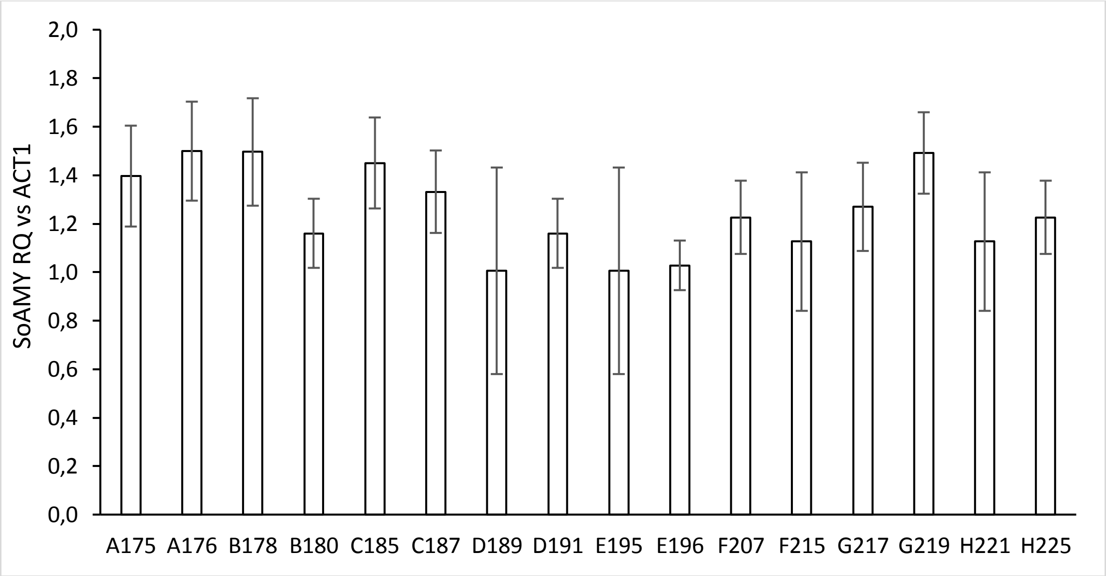

Fig.S2.B. Graphs representing results of ANOVA statistical analysis indicating statistical significance of SP type (p = 0.0000027) and lack of statistical significance of TU order (p = 0.127607) on SoAMY / TlGAMY genes expression (RQ vs actin).

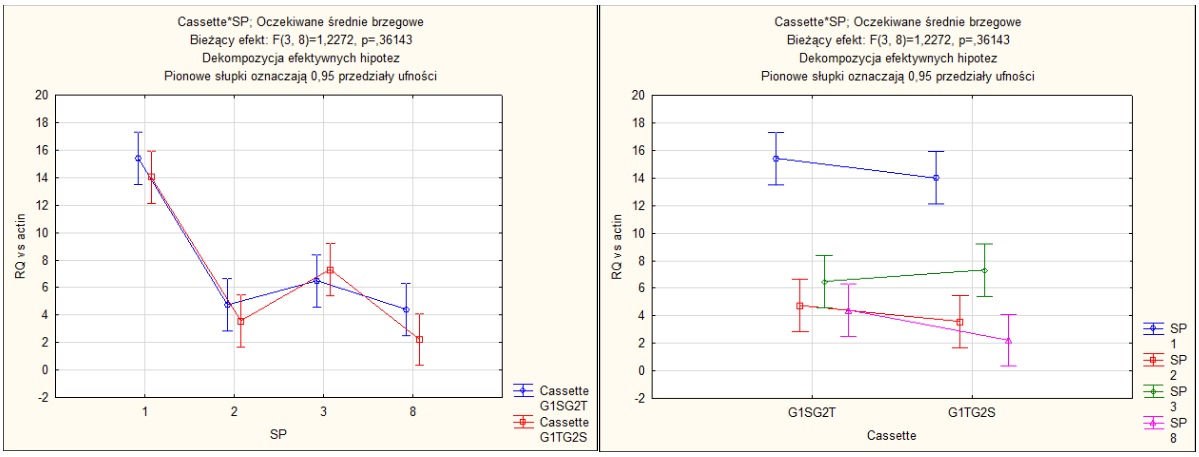

Observation: only SP is a significant parameter affecting expression level of the heterologous genes. Impact of TUs is not statistically significant.

Figure S3 Determination of amylolytic preparation dose

Fig.S3.A Kinetics of the amylolytic preparation depending on the dose – STARCH DEGRADATION

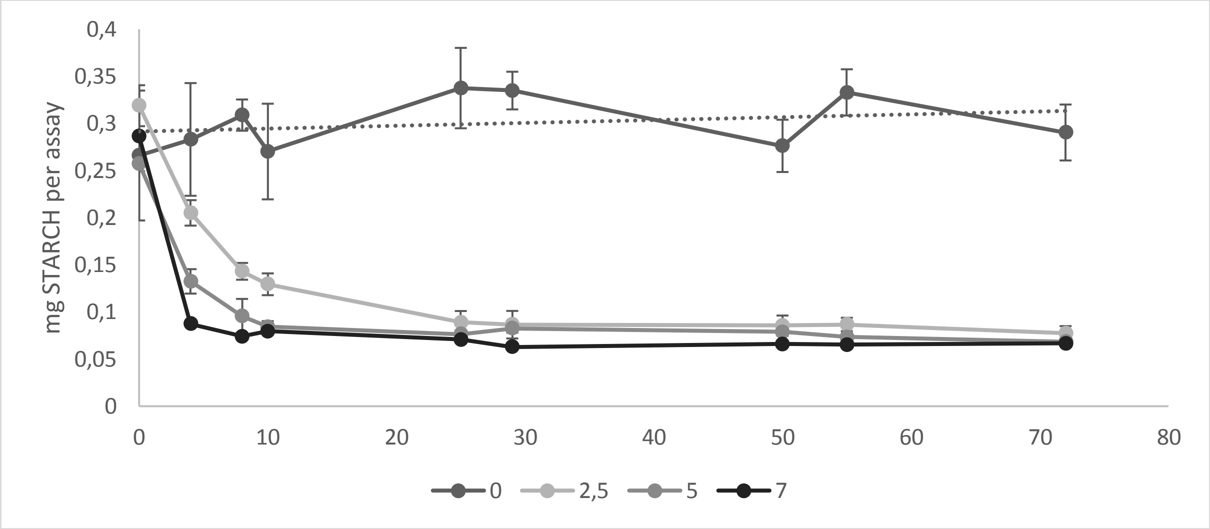

Observation: dose 2.5% results in gradual but complete degradation of starch contained in the adopted medium. Higher doses lead to immediate complete degradation of the substrate.

Fig.S3.B Kinetics of the amylolytic preparation depending on the dose – REDUCING SUGARS RELEASE

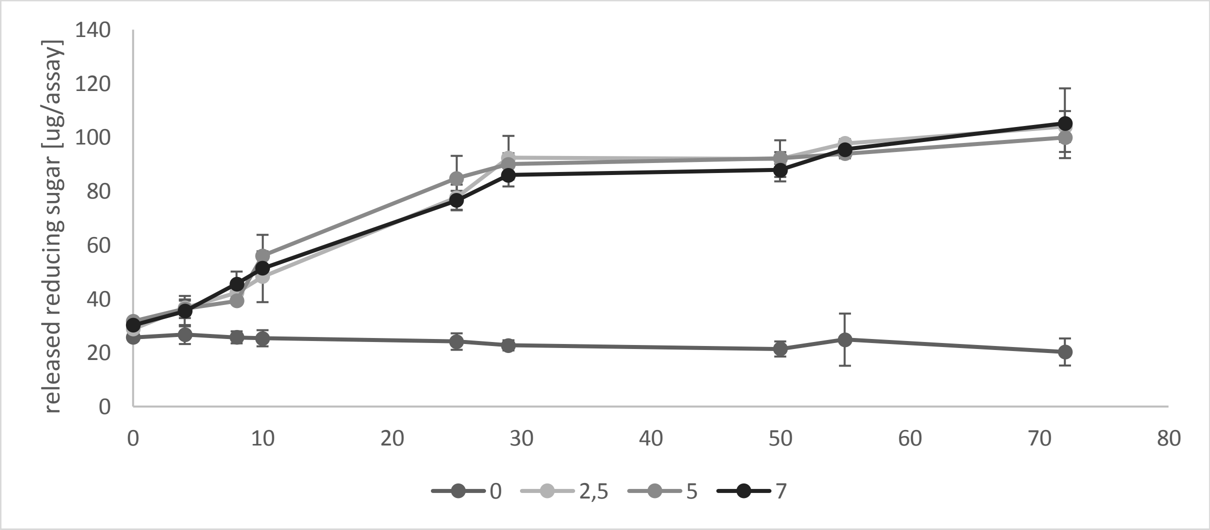

Observation: All doses (2.5 – 7.0 %) result in similar kinetics of reducing sugars release from starch. 2.5% is optimal for the following experiments.

Figure S4 Substrate utilization rate by *Y. lipolytica* F215 strain in batch bioreactor cultures

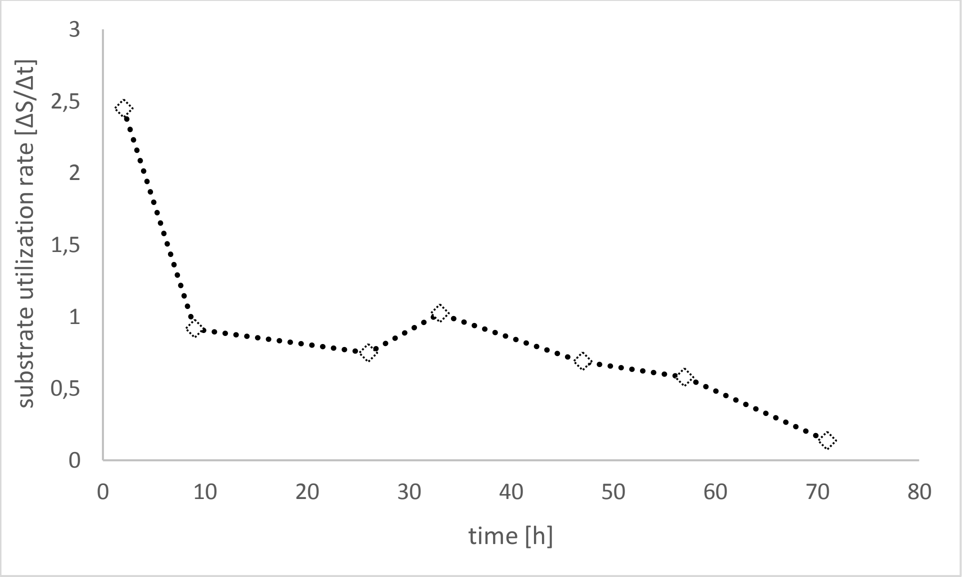

Supplement: Supplementary file 1 — (PDF 501 kb) [file 253_2020_10644_MOESM1_ESM.pdf]
